# Factors affecting *N*-nitrosodimethylamine formation from poly(diallyldimethyl-ammonium chloride) degradation during chloramination

**DOI:** 10.1098/rsos.180025

**Published:** 2018-08-08

**Authors:** Siying Tan, Shaojie Jiang, Xiaoyu Li, Qiuhong Yuan

**Affiliations:** 1 School of Urban Construction and Environmental Engineering, Chongqing University, Chongqing 400044, People's Republic of China; 2 Chongqing Qingze Water Quality Analysis Co., Ltd, Chongqing 401331, People's Republic of China

**Keywords:** poly(diallyldimethylammonium chloride), *N*-nitrosodimethylamine, chloramination, bromide, natural organic matter

## Abstract

Poly(diallyldimethylammonium chloride) (polyDADMAC) has been shown to be an important precursor of the probable human carcinogen *N*-nitrosodimethylamine (NDMA) when in contact with chloramine. In this study, we conducted an orthogonal experiment design to evaluate the effects of pH values, ammonia, bromide, natural organic matter (NOM) and monochloramine dosages on the formation of NDMA from polyDADMAC during chloramination. Meanwhile, single-factor experiments of pH, bromide and NOM prove the results of orthogonal experiment. The results supported that pH was the most critical factor affecting NDMA formation from polyDADMAC during chloramination, and the highest NDMA formation from polyDADMAC occurred at pH near 7 due to released DMA from polyDADMAC degradation and the critical importance of low concentrations of dichloramine in water. In the presence of excess bromide, the NDMA formation was enhanced significantly at all different pH values owing to bromochloramine, which has higher electronegativity of the brominated nitrogen atom than monochloramine or dichloramine. The NDMA formation from polyDADMAC in the presence of NOM was 41.7% lower than NDMA formation in the absence of NOM. The overwhelming majority of NDMA formation from polyDADMAC under simulated conditions was lower than the current advisory levels (i.e. 9 ng l^−1^ in Ontario, 10 ng l^−1^ in California).

## 1. Introduction

*N*-nitrosodimethylamine (NDMA), one of emerging nitrogenous disinfection by-products [1–3], has generated significant concern due to its high probable human carcinogenesis [4,5]. NDMA exhibits 10^−6^ cancer risk level at a concentration as low as 0.7 ng l^−1^ [6]. Thus, US EPA has classified NDMA into the latest drinking water contaminant candidate list 4 (CCL 4) [7]. California Department of Health Care Services has established notification levels for NDMA at 10 ng l^−1^ [8]. Ontario has issued an interim maximum acceptable concentration of 9 ng l^−1^ for NDMA, while in Germany the permissible health-based value for NDMA is equal to 10 ng l^−1^ [9,10].

Owing to the significant health risk of NDMA, previous researches have been conducted in recent years to identify the precursors and formation mechanisms during the process of disinfection. Among NDMA potential precursors, anime-based water treatment polymers, such as poly(epichlorohydrin dimethylamine) (polyamine), poly(diallyldimethylammonium chloride) (polyDADMAC) and cationic polyacrylamide, have received considerable attention due to their usage in water and wastewater treatment [11–15]. Compared with other amine-based water treatment polymers, polyDADMAC is widely used as coagulant and flocculent aid for water treatment. The structure of polyDADMAC is shown in electronic supplementary material, figure S1. Park *et al*. proposed that NDMA formation from polyDADMAC has two steps: (1) the degradation of its quaternary ammonium ring group and DMA release; (2) the reaction between released DMA and dichloramine to generate chlorinated unsymmetrical dimethylhydrazine (Cl-UDMH) intermediate, followed by subsequent oxidation of Cl-UDMH by dissolved oxygen [16]. Zeng *et al*. [17] classified the precursors of NDMA from untreated polyDADMAC into three pools, free secondary or tertiary amines, polymer-bound tertiary amines and polymer-bound quaternary ammonium groups serving as the intended functional groups within polymers, and their average contributions of NDMA formation were 16 ± 2%, 25 ± 5%, and 57 ± 11%, respectively.

Many factors affect the NDMA formation from polyDADMAC, including pH, bromide, natural organic matter (NOM), ammonia and oxidant dosage. Park *et al*. [16] reported that the NDMA formation from polyDADMAC degradation during chloramination was comparable from pH 6 to 8, and lower at pH 5 or 9, due to the degradation of polyDADMAC and the importance of dichloramine (NHCl_2_) [18,19]. Bromide is a frequent component of natural waters and also influenced by pH value in water. With an excess of bromide, monochloramine (NH_2_Cl) is not stable and degrades rapidly. The overall reactions are described as (1.1) and (1.2) [20–22]. Luh & Marinas [23] evaluated the effect of bromide on NDMA formation from DMA at various pH values and demonstrated that the presence of bromide enhanced NDMA formation at the relatively high pH values of 8 and 9 after 24 h of reaction time, whereas at relatively low to neutral pH (6 to 7), the presence of bromide resulted in lower NDMA formation when compared to results obtained in the absence of bromide. However, prior researches used only DMA as model precursor to investigate the effect of bromide on NDMA formation. The effect of bromide on NDMA formation from polyDADMAC is still uncertain.

1.1 $$2\text{N}{\text{H}}_{2}\text{Cl}+{\text{H}}^{+}+\text{B}{\text{r}}^{-}\to \text{NHBrCl}+{\text{NH}}_{4}^{+}+\text{C}{\text{l}}^{-}$$

1.2 $$\text{NHBrCl}+\text{N}{\text{H}}_{2}\text{Cl}\to {\text{N}}_{2}+\text{B}{\text{r}}^{-}+2\text{C}{\text{l}}^{-}+3{\text{H}}^{+}.$$

NOM widely exists in drinking water treatment [24,25]. Dissolved organic nitrogen (DON), a small portion of NOM by weight (0.5–10%) [26], was considered as the precursor of NDMA, but application of monochloramine to organic matter isolates indicated no clear correlation between NDMA formation and DON [27]. Generally, the hydrophilic fractions of NOM tend to form more NDMA than hydrophobic fractions, and basic fractions tend to form more NDMA than acidic fractions when normalized to a carbon basis [28]. But accounting for the contribution of each fraction of the total dissolved organic carbon in river water, the low reactivity of the hydrophobic acid fraction still accounted for nearly 70% of the NDMA formation potential [28]. Some 90% of NOM was humic acids, troublesome materials in drinking water treatment [29]. Polymeric flocculants, such as polyDADMAC, are commonly used by water utilities to enhance the removal of particles and NOM. Even though NOM and polyDADMAC were confirmed as NDMA precursors, there are few reports on the effect of NOM on polyDADMAC forming NDMA. Besides the three factors mentioned before, the sequence of chlorine and ammonia addition and free chlorine to ammonia (Cl_2_ : NH_3_) ratio for *in situ* chloramines formation have been discussed as important factors affecting NDMA formation. Schreiber & Mitch [18,30] disclosed the important role of dichloramine, forming in locally high Cl_2_ : NH_3_ when free chlorine was added to a well-mixed ammonium solution. The authors proposed a NDMA pathway that DMA primarily reacted with dichloramine to form Cl-UDMH, which was further oxidized by dissolved oxygen. A more recent study evaluated the effect of *in situ* chloramination and supported that dichloramine formation was the critical factor affecting NDMA formation from polyDADMAC and polyamine during *in situ* chloramination [19]. Among these researches to identify NDMA precursors, extremely high monochloramine dosage (2 mM) was used in researches [13,31]. Such high oxidant dose could not represent the situation in practical water treatment. The investigation of the impact of monochloramine dose (4–13 mg l^−1^ as Cl_2_ l^−1^) showed NDMA formation from both polyamines and polyDADMAC increased with monochloramine dose [16]. Even though it is true that polyDADMAC is one of the NDMA precursors in the previous researches, it is still controversial as to whether or not polyDADMAC leads to NDMA at levels of concern in real water treatment oxidant dose.

To date, limited studies have assessed the effects of those aforementioned factors on NDMA formation from polyDADMAC. To better understand NDMA formation from polyDADMAC degradation, the first objective of this study is to conduct an orthogonal experimental design to investigate the effects of pH, ammonia, bromide, NOM and oxidant dose on NDMA formation from polyDADMAC. The second objective is to determine the effect of bromide on NDMA formation from polyDADMAC degradation in waters containing monochloramine at various pH values. The third objective is to investigate whether or not polyDADMAC leads to NDMA at levels of concern in reaction conditions which are more likely encountered in water and wastewater treatment plants.

## 2. Material and methods

### 2.1. Materials

Nitrosamines mix (99%), *N*-nitrosodimethylamine-d6 (94%, NDMA-d6) and *N*-nitrosodi-*n*-propylamine-d14 (98%, NDPA-d14) were from Accustandard and used as standards without further purification. Deionized water (DI), produced from a Milli-Q Reference water purifying system, was used for all experiments. PolyDADMAC (40.0 wt% aqueous solutions, Mw 10 000–20 000, 60–80 cp) was obtained from Aladdin and used as received. Fresh stock solution (1000 mg l^−1^ as active ingredient) of polymer was made every time for each experiment. Solid-phase extraction (SPE) cartridges (model 26032) were from Restek for EPA Method 521 [32]. The details of other materials are listed in electronic supplementary material, text S1.

### 2.2. Experimental procedures

All glassware for NDMA formation experiments was baked at 350°C for 3 h prior to use, with the exception of volumetric glassware that was rinsed with acetone. Monochloramine solutions were prepared fresh every time as described in Mitch's research [33] and by dissolving ammonium chloride in DI adjusted to pH 9.6 with sodium hydroxide and chilled to 5°C. Sodium hypochlorite was added slowly to a rapidly stirred solution at a molar ratio of at least 1 : 1.2 for hypochlorite to ammonia. The adjustment of the pH to around 9.6 was used to minimize the disproportionation of monochloramine to dichloramine. Maintaining a slight excess of ammonia and slow addition of hypochlorite could reduce the potential for breakpoint chlorination and result from localized excesses of hypochlorite due to poor mixing. The resulting solutions were standardized by using spectrophotometric *N*,*N*′-diethyl-*p*-phenylenediamine method [34].

A baseline monochloramine dose of 10 mg l^−1^ was applied with 10 mg l^−1^ as active ingredient of fresh polyDADMAC buffered (10 mM phosphate buffer) at pH 7, and incubated in the dark at 25°C for 24 h. Afterward, the reactions were quenched by sodium hyposulfite before NDMA and DMA analysis. The NDMA concentration was measured at the end of the reaction. Humic acid was chosen to represent NOM. The process of jar test is listed in electronic supplementary material, text S2. For all the experiments, matrix and reagent controls as well as duplicate experiments were conducted for each condition.

An orthogonal experimental design was carried out to evaluate the effects of pH values, ammonia, bromide ion concentration, NOM concentration and monochloramine dosages. The test values of each factor are shown in the electronic supplementary material, table S1. According to test values, L_25_(5^6^) was carried out for orthogonal experiment.

### 2.3. Analytical methods

#### 2.3.1. *N*-nitrosodimethylamine analytical method

The SPE producer was following EPA Method 521 using coconut charcoal tubes followed by GC/MS analysis [32]. In brief, NDMA-d6 was added to samples as a surrogate before SPE. SPE cartridges were conditioned using dichloromethane, methanol, and DI before samples were passed through the cartridges. Cartridges were then dried to air and eluted with dichloromethane. Effluents were passed through a sodium sulfate SPE tube to remove residual water and concentrated under a gentle stream of ultrahigh purity nitrogen gas to 1 ml. NDPA-d14 was added to extracts as an internal standard, and analysed using a Thermo Scientific DSQ II Single Quadrupole GC/MS with a DB-624 (60 m × 250 µm × 0.25 µm). The GC oven temperatures were held at 35°C for 1 min, ramped at 35°C min^−1^ to 200°C, and held for 3 min, then ramped at 10°C min^−1^ to 240°C, with a final hold at 14 min. NDMA and NDMA-d6 were quantified by selective ion monitoring using *m/z* 74 and 80. To evaluate method detection limit (MDL), calibration-standard solutions (10 ng l^−1^) were made in triplicate and extracted. Estimated MDL was calculated as 3 × standard deviation of three independently spiked samples. The detection limit of this method for NDMA was around 3.1 ng l^−1^.

#### 2.3.2. Dimethylamine analytical method

The DMA analytical method was a modified version of the traditional method derivatizated with benzenesulfonyl chloride from Sacher *et al*. [35]. In brief, 900 µl of 400 g l^−1^ aqueous sodium hydroxide solution and 100 µl benzenesulfonyl chloride were added to samples (10 ml). The samples were shaken vigorously until benzenesulfonyl chloride dissolved and put in a water bath at 80°C. Subsequently, the samples were cooled down with ice water, and acidified with 2 ml acetic acid buffer to pH 5.5, extracted in 1.5 ml dichloromethane, and analysed by GC/FID (Aglient 7890B) with an HP-5 (30 m  × 320 µm × 0.25 µm). The GC oven temperatures began at 100°C for 3 min, then ramped at 10°C min^−1^ to 240°C. To evaluate MDL, calibration-standard solutions (5 µg l^−1^) were made in triplicate and derivatizated. Estimated MDL was calculated as 3 × standard deviation of three independently spiked samples. The detection limit of this method for DMA was around 0.5 µg l^−1^.

### 2.4. Poly(diallyldimethylammonium chloride) structural analysis by Fourier transform infrared spectroscopy

To investigate polyDADMAC structural change by chloramines with bromide, type A (5 g l^−1^ as active ingredient of polyDADMAC only), type B (5 g l^−1^ as active ingredient of polyDADMAC and 1 g l^−1^ preformed monochloramine) and type C (5 g l^−1^ as active ingredient of polyDADMAC and 1 g l^−1^ preformed monochloramine with 2 mM bromide) were reacted respectively at pH 7.5 for 24 h at room temperature. Afterward, the polymers were dried and analysed by Fourier transform infrared (FTIR) spectroscopy.

## 3. Results and discussion

### 3.1. Orthogonal experimental design

The results of table 1 show the range of NDMA formation from polyDADMAC differed from 147.41 to 1395.36 ng l^−1^. The maximal NDMA formation occurred when pH value, the concentration of ammonia, the concentration of bromide ion, the concentration of NOM and the dose of monochloramine were 8, 0 mg l^−1^, 0.2 mM, 0.25 mg l^−1^ and 10 mg l^−1^ as Cl_2_ l^−1^. The results of range analysis show that the influence of polyDADMAC forming NDMA was sorted from highest to lowest: pH, bromide, NOM, ammonia and the dose of monochloramine (table 1). According to analysis of variance (electronic supplementary material, table S2), pH is the most significant factor affecting polyDADMAC forming NDMA.

**Table 1.** The results and analysis of average values of orthogonal experimental design.

test	pH	N-NH_4_^+^	Br^−^	NOM	NH_2_Cl	blank	NDMA (ng l^−1^)	DMA (μg l^−1^)
no.0^a^							<3	4.92
no.1	1	1	1	1	1	1	244.54	12.53
no.2	1	2	2	2	2	2	279.10	24.98
no.3	1	3	3	3	3	3	374.68	17.84
no.4	1	4	4	4	4	4	612.27	13.88
no.5	1	5	5	5	5	5	400.57	12.62
no.6	2	1	2	3	4	5	325.04	17.92
no.7	2	2	3	4	5	1	735.36	18.33
no.8	2	3	4	5	1	2	689.82	10.28
no.9	2	4	5	1	2	3	887.97	11.31
no.10	2	5	1	2	3	4	406.97	11.67
no.11	3	1	3	5	2	4	658.92	17.52
no.12	3	2	4	1	3	5	1022.15	15.43
no.13	3	3	5	2	4	1	1313.98	16.02
no.14	3	4	1	3	5	2	516.06	11.27
no.15	3	5	2	4	1	3	708.28	11.74
no.16	4	1	4	2	5	3	1395.36	17.07
no.17	4	2	5	3	1	4	673.16	10.59
no.18	4	3	1	4	2	5	436.45	11.02
no.19	4	4	2	5	3	1	297.75	13.04
no.20	4	5	3	1	4	2	465.17	12.47
no.21	5	1	5	4	3	2	405.05	12.02
no.22	5	2	1	5	4	3	164.71	11.98
no.23	5	3	2	1	5	4	446.35	11.76
no.24	5	4	3	2	1	5	228.64	11.45
no.25	5	5	4	3	2	1	147.41	12.22
$\bar{\text{NDMA}}$^b^	I	382.23	605.78	353.75	613.24	508.89	547.81	
	II	609.03	574.90	411.30	724.81	481.97	472.51	
	III	843.88	652.26	493.55	407.27	501.32	666.03	
	IV	653.58	508.54	773.40	579.48	576.23	559.53	
	V	278.43	425.68	736.15	442.35	698.78	482.57	
	range	565.45	226.58	419.66	317.54	216.77	193.52	
$\bar{\text{DMA}}$^b^	I	16.37	15.41	11.69	12.70	11.32	14.43	
	II	13.90	16.26	15.89	16.24	15.41	14.64	
	III	14.39	13.38	15.52	13.97	14.00	13.73	
	IV	12.84	12.19	13.78	13.40	14.45	13.08	
	V	11.89	12.14	12.52	13.09	14.21	13.69	
	range	4.48	4.12	4.19	3.54	4.10	1.55	

^a^Measurement of DMA and NDMA in 10 mg l^−1^ polyDADMAC solution buffed at pH 7 in the dark at 25°C for 24 h.

^b^Average values of each level.

The range of residual DMA concentration differed from 10.28 to 24.98 µg l^−1^ (table 1). The maximal DMA residual occurred when pH value, the concentration of ammonia, the concentration of bromide ion, the concentration of NOM and the dose of monochloramine were 5, 0.1 mg l^−1^, 0.05 mM, 0.25 mg l^−1^ and 4 mg l^−1^ as Cl_2_ l^−1^. The influence of DMA residual concentration was sorted from highest to lowest: pH, bromide, ammonia, the dose of monochloramine and NOM. NOM was the only factor that was not significant for DMA residual concentration (electronic supplementary material, table S2), which indicated NOM did not impact polyDADMAC degradation to DMA.

**Figure 1.**
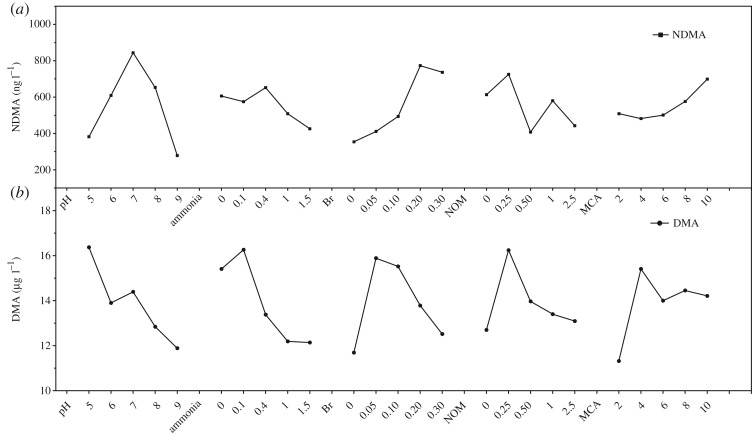
Effect diagram of pH, ammonia (mg l^−1^), bromide (mM), NOM (mg l^−1^) and monochloramine (MCA, mg l^−1^ as Cl_2_ l^−1^) on NDMA formation (*a*) and DMA residual concentration (*b*) from polyDADMAC. Ten mg l^−1^ as active ingredient of polyDADMAC was reacted with preformed monochloramine for 24 h at 25°C.

The trends of NDMA formation and DMA residual from polyDADMAC from the five factors are shown in figure 1. NDMA formation increased with pH values, maximized at pH 7, afterward decreased. In the presence of ammonia, NDMA formation decreased slightly first, reached the highest value at 0.4 mg l^−1^ ammonia, afterward decreased. Bromide significantly enhanced NDMA formation from polyDADMAC, whereas the presence of NOM caused the decrease of NDMA formation (figure 1*a*). DMA residual concentration decreased with the pH values. DMA residual increased with ammonia first, afterwards decreased. The effect of bromide, NOM and the dose of monochloramine had similar trends (figure 1*b*). The NDMA formation increased, afterward decreased with the increase of concentration.

According to range analysis (table 1) and the analysis of variance (electronic supplementary material, table S2), pH values and bromide were the two most significant factors that affect NDMA formation from polyDADMAC degradation. Besides, NOM was the third significant factor; also, polyDADMAC was used to remove the NOM of drinking treatment. Thus, pH, bromide and NOM were chosen to investigate the effects on NDMA formation from polyDADMAC in the next sections.

### 3.2. Effect of pH

**Figure 2.**
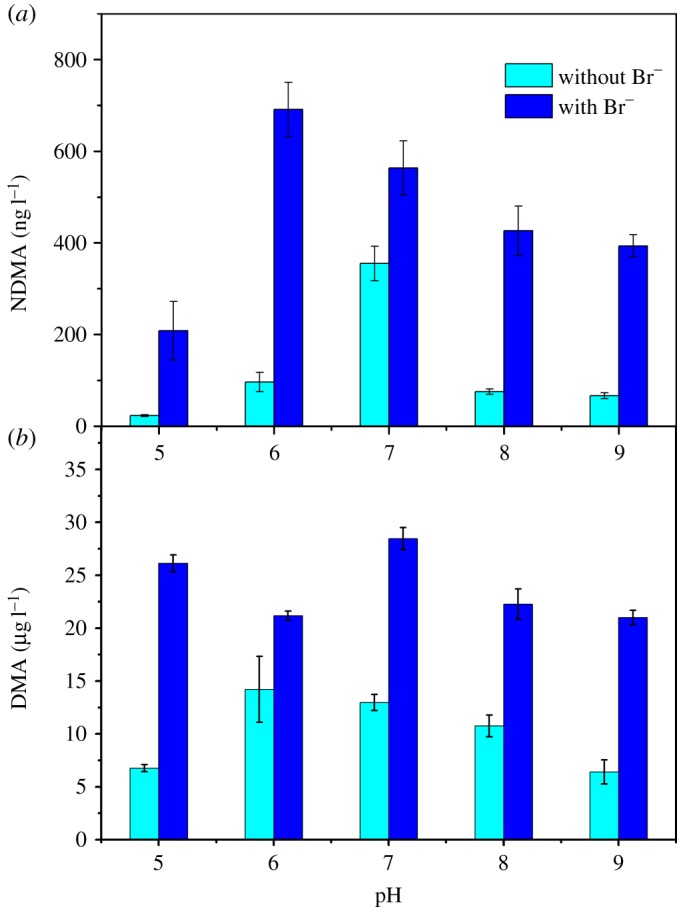
NDMA formation (*a*) and DMA residual concentration (*b*) in the absence of and presence of (0.4 mM) bromide from polyDADMAC. Ten mg l^−1^ as active ingredient of polyDADMAC was reacted with 10 mg l^−1^ as Cl_2_ l^−1^ of preformed monochloramine for 24 h at 25°C with or without 0.4 mM bromide at pH 5, 6, 7, 8 and 9 (10 mM phosphate buffer). Error bars represent one standard deviation of the measurement derived from the standard curve.

Figure 2 depicts NDMA formation and DMA residual concentration after dosing with preformed monochloramine at 10 mg l^−1^ as Cl_2_ l^−1^ in 10 mg l^−1^ as active ingredient of polyDADMAC solutions at different pH values with or without 0.4 mM bromide. The chloramines residual after 24 h as a function of pH without bromide was measured (electronic supplementary material, figure S2). NDMA formation was maximized at pH 7 for polyDADMAC in absence of bromide. Solution pH affects the NDMA formation in the following two ways.

#### 3.2.1. pH affects dimethylamine from poly(diallyldimethylammonium chloride) degradation

Figure 2*b* presents the residual DMA concentration after the reaction with or without bromide. In the absence of bromide, the concentration of DMA after reaction (from 6.76 to 14.22 µg l^−1^) was higher than the initial residual DMA concentration (i.e. 4.92 µg l^−1^). The results indicated the free tertiary amines, polymer-bound tertiary amines and polymer-bound quaternary ammonium groups of polyDADMAC solution degraded and released DMA, which is consistent with previous research [16]. The DMA residual concentration was much higher at pH 6 and 7 than pH 5, 8, or 9 without bromide, which suggested polyDADMAC degraded more DMA at pH 6 and 7. But the pH value of the maximum residual DMA (i.e. pH 6) differed from that of maximum NDMA formation (i.e. pH 7). The residual DMA reflected the sum of initial DMA and newly released DMA subtracted by DMA loss due to the reaction with monochloramine. The neutral pH was expected to release more DMA; however, the larger amount of DMA loss would form NDMA at neutral pH. A combination of the above two phenomena likely resulted in the highest DMA residual at pH 6.

#### 3.2.2. pH affects the speciation of chloramines

3.1 $$2\text{N}{\text{H}}_{2}\text{Cl}+{\text{H}}^{+}\overset{k}{\to }\text{NHC}{\text{l}}_{2}{\text{+ NH}}_{4}^{+}\;(k=6.9\times 10^{3}\;{\text{M}}^{-2}\;{\text{s}}^{-1})\;[36].$$

The importance of dichloramine has been proved for NDMA formation [18]. Owing to reaction (3.1), NH_2_Cl and NHCl_2_ coexist in the water. As shown in electronic supplementary material figure S2, the average percentages of NHCl_2_ in the total oxidant concentration were 69%, 20% and 4.6% at pH 5, 6 and 7, respectively, which was consistent with previous results [37]. NHCl_2_ could be negligible above pH 8. Thus, NDMA formation at intermediate pH was higher than that above 8. Also, NDMA formation is the most favourable when unprotonated DMA reacts with NHCl_2_, whereas unprotonated DMA is more abundant at higher pH [18]. Therefore, the NDMA formation from polyDADMAC below 6 was lower than that at pH 7.

Based on above discussion, the highest NDMA formation occurring at neutral pH value could be explained by DMA degraded from polyDADMAC and the critical importance of low concentrations of NHCl_2_.

### 3.3. Effect of bromide on *N*-nitrosodimethylamine formation from poly(diallyldimethylammonium chloride) during chloramination

#### 3.3.1. Effect of different bromide ion concentration

**Figure 3.**
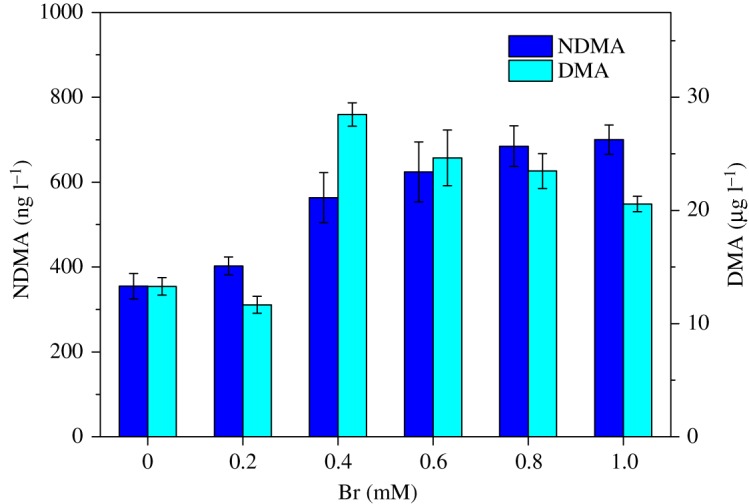
Effect of bromide on NDMA formation from polyDADMAC. Ten mg l^−1^ as active ingredient of polyDADMAC was reacted with 10 mg l^−1^ as Cl_2_ l^−1^ of preformed monochloramine with different bromide ion concentration for 24 h at 25°C at pH 7 (10 mM phosphate buffer). Error bars represent one standard deviation of the measurement derived from the standard curve.

**Figure 4.**
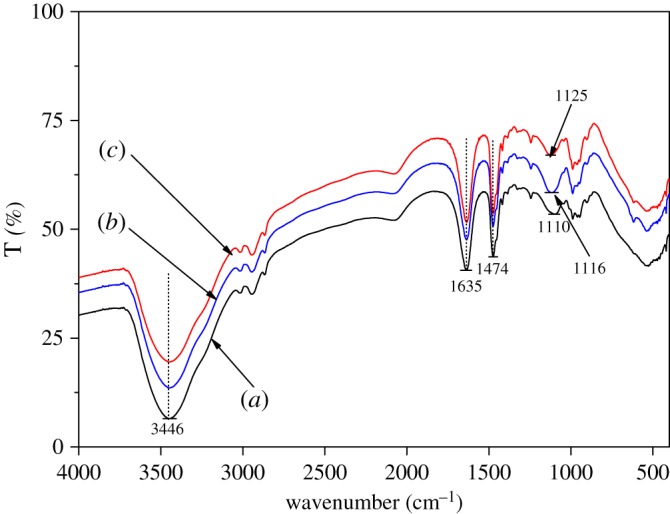
FTIR spectra of (*a*) polyDADMAC only, (*b*) polyDADMAC and monochloramine and (*c*) polyDADMAC and monochloramine with bromide.

**Figure 5.**
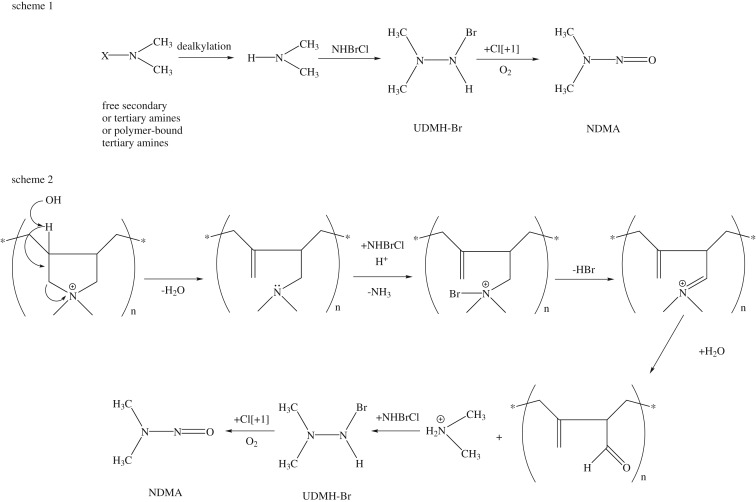
Proposed degradation pathways for polyDADMAC forming NDMA during chloramine in the presence of bromide.

Figure 3 presents NDMA formation and residual DMA concentration after dosing with preformed monochloramine at 10 mg l^−1^ as Cl_2_ l^−1^ in 10 mg l^−1^ as active ingredient of polyDADMAC at different bromide ion concentration. In the presence of bromide, the NDMA formation from polyDADMAC was increased with bromide ion concentration, and reached a plateau after 0.4 mM. The enhanced NDMA formation is due to the formation of bromochloramine (NHBrCl). In the presence of bromide, reactions (3.2)–(3.4) happen in the chloramine solution to form NHBrCl [38]. Compared with NH_2_Cl or NHCl_2_, NHBrCl had higher electronegativity of the brominated nitrogen atom [20]. To investigate polyDADMAC structural changes by NHBrCl, FTIR was conducted. Much higher polyDADMAC usage, monochloramine concentrations and bromide ion concentration were used to meet the detection limits of FTIR. Figure 4 illustrates the spectra of polyDADMAC only, polyDADMAC with monochloramine and polyDADMAC with monochloramine and bromide. The trends of polyDADMAC of the three conditions were similar. In the spectra, 3446 cm^−1^, 1635 cm^−1^and 1474 cm^−1^ were observed for –OH stretch, ─CH asymmetrical bending and –CH in-plane bending, respectively. The peaks of C–N stretching vibration were at 1110 cm^−1^, 1116 cm^−1^ and 1125 cm^−1^ under the conditions of polyDADMAC only, polyDADMAC with monochloramine and polyDADMAC with monochloramine and bromide, respectively. This shift might have been caused by inductive effects of electronegative substituents (NH_2_Cl and NHBrCl). NHBrCl had the highest electronegativity and caused the highest increase of wavenumber. The peak intensity of C–N stretching vibration from highest to lowest was the conditions of polyDADMAC only, polyDADMAC with monochloramine and polyDADMAC with monochloramine and bromide. The decrease of peak intensity can be considered as evidence of ring opening of polyDADMAC after chloramination and bromochloramine. Considering the observation of FTIR experiment, we postulated the pathway of NDMA formation from polyDADMAC in the presence of bromide. The free secondary, tertiary amines or polymer-bound tertiary amines of the polyDADMAC solutions undergo dealkylation to release DMA. DMA reacts with NHBrCl to form a hypothetical Br-UDMH similar to the formation of Cl-UDMH from NHCl_2_ (figure 5, scheme 1). PolyDADMAC, the quaternary ammonium compound, first undergoes a Hofmann elimination [16] and followed by attack of NHBrCl on the resulting tertiary amine (figure 5, scheme 2).

3.2 $$\begin{array}{rl} & \text{N}{\text{H}}_{2}\text{Cl}+{\text{H}}^{+}⇌\text{N}{\text{H}}_{3}\text{C}{\text{l}}^{+}\;K_{1}=28{\text{M}}^{-1}{\text{l}}^{3}, \end{array}$$

3.3 $$\begin{array}{rl} & \text{N}{\text{H}}_{3}\text{C}{\text{l}}^{+}+\text{B}{\text{r}}^{-}\to \text{N}{\text{H}}_{3}\text{B}{\text{r}}^{+}+\text{C}{\text{l}}^{-}\;K_{2}=1.8\times 10^{8}{\text{M}}^{-1}{\text{h}}^{-1} \end{array}$$

3.4 $$\begin{array}{rl} \;\text{and}\; & \text{N}{\text{H}}_{3}\text{B}{\text{r}}^{+}+\text{N}{\text{H}}_{2}\text{Cl}\overset{\mathrm{fast}}{\to }\text{NHClBr}+{\text{NH}}_{4}^{+}. \end{array}$$

The trend of residual DMA was more complex than NDMA formation from polyDADMAC. The residual DMA concentration in the absence of bromide was comparable to the concentration in the presence of bromide when bromide ion concentration was less than 0.4 mM (figure 3). Whereas, the residual DMA concentration when bromide ion concentration was more than 0.4 mM was twice or thrice as when bromide ion concentration was less than 0.4 mM. The results suggested that bromide has negligible impact for polyDADMAC degradation when bromide ion concentration was less than 0.4 mM, whereas the existence of bromide enhanced the residual DMA concentration from polyDADMAC degradation.

#### 3.3.2. Effect of bromide and pH

The NDMA formation in figure 2*a* has a similar trend to that shown in figure 1*a*. Bromide enhanced the NDMA formation from polyDADMAC at all pH values. There are two explanations: (1) more DMA degraded from polyDADMAC, which could be proved by the trend of residual DMA concentration in figure 2*b* (in the presence of bromide, residual DMA concentration at all pH values was higher than that in absence of bromide); (2) the formation of NHBrCl, which has already been discussed in §3.3.1. The highest NDMA formation occurred at pH 7 in absence of bromide while the maximum NDMA formation was at pH 6 in presence of bromide. The determining rate of NHBrCl formation was the formation of NH_3_Cl^+^ (reaction (3.2)). NH_3_Cl^+^ favoured the acid condition, so more NHBrCl formed [38]. With the increase of pH, dibromamine (NHBr_2_) was the predominant component in chloramine solution with bromide when pH was higher than 8. However, NHBr_2_ was not an effective factor of NDMA formation [20,23]. In previous research, Luh *et al*. [23] found that 0.0005 mM DMA reacted with 0.2 mM NH_2_Cl to form NDMA in the presence of 0.4 mM bromide at pH 6, 6.3, 6.6, 7, 8 and 9. The presence of bromide enhanced NDMA formation at the relatively high pH values of 8 and 9, which was consistent with our study. However, at relatively low to neutral pH (6 to 7), the presence of bromide resulted in lower NDMA formation as compared to results obtained in the absence of bromide. The reason could be that polyDADMAC might degrade other intermediates which were easier to react with NHBrCl or NHCl_2_.

Residual DMA concentration was enhanced by bromide but not affected by different pH values (figure 2*b*). The residual DMA concentration ranged from 21.0 to 28.5 µg l^−1^. The lowest residual DMA concentration occurred at pH 6. Residual DMA concentration represented the initial DMA concentration of polyDADMAC plus the DMA concentration from polyDADMAC degradation minus DMA loss in the following reaction; thus, more released DMA reacted with mixed dihaloamine bromochloramine at pH 6.

### 3.4. Effect of natural organic matter

**Figure 6.**
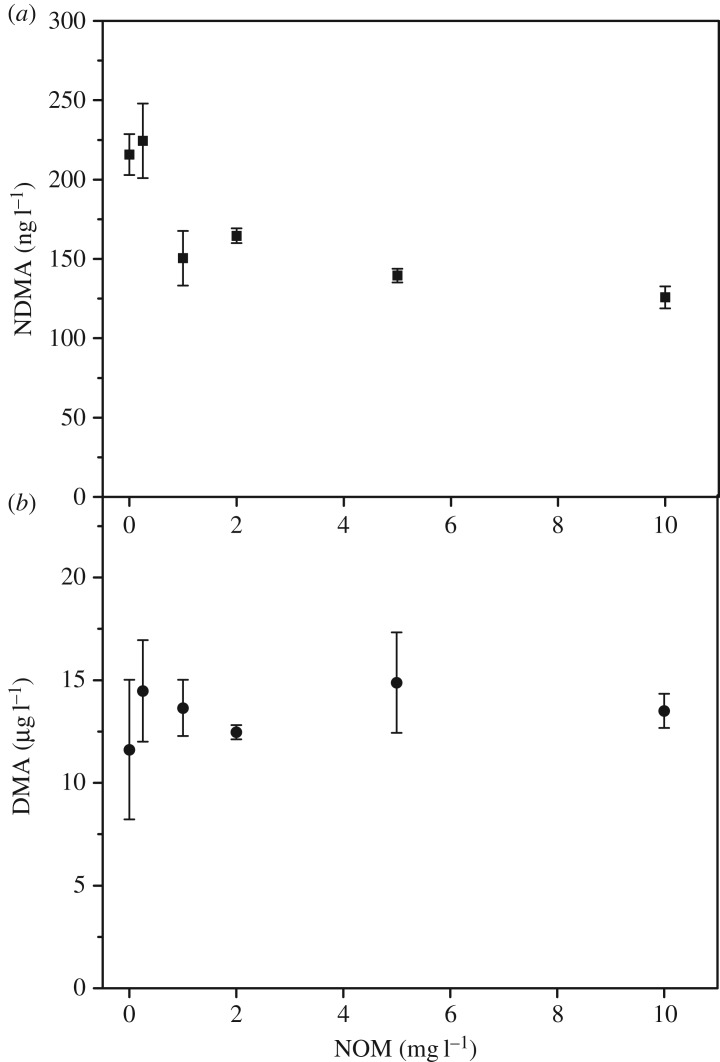
Effect of natural organic matter (NOM) concentration on NDMA formation (*a*) and DMA residual concentration (*b*) from polyDADMAC. Ten mg l^−1^ as active ingredient of polyDADMAC was reacted with 10 mg l^−1^ as Cl_2_ l^−1^ of preformed monochloramine for 24 h at 25°C at pH 7 (10 mM phosphate buffer). Error bars represent one standard deviation of the measurement derived from the standard curve.

Figure 6 illustrates the NDMA formation and residual DMA concentration from polyDADMAC degradation at different NOM concentration. In the presence of NOM, NDMA formation was less than in the absence of NOM, whereas residual DMA concentration stayed constant, which was consistent with the results of orthogonal experiments (figure 1). These results indicate that the presence of NOM did not affect DMA released from polymer-bound tertiary amines and polymer-bound quaternary ammonium groups of polyDADMAC solution, but hindered the reaction between DMA and NHCl_2_ to form NDMA. To assess the effect of NOM, the experiments were conducted and results are given in table 2. Of note, 10 mg l^−1^ polyDADMAC and 10 mg l^−1^ NOM formed 215.78 and 15.22 ng l^−1^ NDMA, respectively, which indicated polyDADMAC poses a bigger threat than NOM in drinking water treatment process similar to previous research [26]. The results of experiment A and C showed NDMA formation in the presence of NOM decreased 41.7% compared to that in the absence of NOM. The hypothesis of the decrease was that NOM competes with DMA for the oxidant. To verify this hypothesis, experiments E and F were conducted. Of note, 50 µg l^−1^ DMA of experiments E and F were left 62.0% and 74.0%, respectively, and NDMA formation decreased 35.3% in the presence of NOM compared to in the absence of NOM, which was similar to the NDMA formation from polyDADMAC. Those results verified the hypothesis that the reaction between NOM and monochloramine reduced the possibility that DMA was oxidized by monochloramine. The existence of NOM reduced NDMA formation from DMA during chloramination, because (1) NOM competed with DMA for oxidant to form other disinfection by-products and (2) a complexation happened between DMA and polar charged NOM hindering NDMA formation [39]. Experiment C only tested the NDMA formation potential of the coexistence of polyDADMAC and NOM. However, in the practical process of drinking water treatment, polyDADMAC was used to remove NOM through coagulation and flocculation. To investigate whether coagulation between NOM and polyDADMAC affected NDMA formation, the jar test was conducted to model the coagulation and supernatant was chosen to incubate to NDMA, namely, experiment F. The details of the jar test are given in electronic supplementary material, text S2. Compared with experiment C, the NDMA formation of experiment F decreased only 11.9% and the residual DMA concentration was comparable, which indicated that coagulation has limited effect on polyDADMAC degradation, and polyDADMAC still posed the threat of NDMA formation even after coagulation.

**Table 2.** Influence of NOM on NDMA formation and residual DMA concentration from polyDADMAC with 10 mg Cl_2_ l^−1^ of monochloramine for 24 h at pH 7 with 10 mM phosphate buffer and 25°C.

experiment	polyDADMAC (mg l^−1^ as a.i.)	NOM (mg l^−1^)	DMA (μg l^−1^)	NDMA (ng l^−1^)	DMA(μg l^−1^)^a^
A	10	0	0	215.78 ± 2.12	11.62 ± 1.21
B	0	10	0	15.22 ± 1.35	1.67 ± 0.56
C	10	10	0	125.80 ± 4.31	13.50 ± 3.87
D	0	0	50	1375.03 ± 7.90	31.06 ± 1.61
E	0	10	50	889.34 ± 3.21	36.75 ± 2.83
F^b^	10	10	0	111.87 ± 4.07	11.70 ± 1.09

a.i., active ingredient.

^a^Residual DMA in solution after reaction.

^b^PolyDADMAC and NOM coagulated by jar test and supernatant was used to test.

In order to get better understanding of the situations in practice, we conducted the experiments in table 3 which were more representative of situations encountered in water and wastewater treatment plants. NOM and bromide were added in DI, and coagulated with polyDADMAC by jar test. After coagulation, the solution was dosed with preformed monochloramine at 4 mg l^−1^ as Cl_2_ l^−1^ for 2 h. The NDMA formation was tested after reaction. As shown in table 3, the NDMA formation of water samples 2, 3, 7, 8, 9 and 10 was less than MDL, because only 1 mg l^−1^ polyDADMAC was added in water sample. The amount of polyDADMAC was the main factor of NDMA formation. Compared with the results of figure 6 and table 2, NDMA formation of water samples 4, 5, 11 and 12 had different trends (table 3). NDMA formation was reduced in the presence of NOM in figure 6 and table 2, while the presence of NOM increased slightly NDMA formation in the simulated condition (table 3). The reason is that NOM was removed by coagulation, but ample amount of polyDADMAC still existed in the water and reacted with monochloramine. The results of water sample 4 versus water sample 7, sample 11 versus sample 15 versus water sample 16, and sample 12 versus water sample 13 versus water sample 14 indicated that NDMA formation was increased with the bromide ion concentration, which was consistent with the results of §3.3. Overall, the results of table 3 strongly suggested that the overwhelming majority of NDMA formation of polyDADMAC under simulated conditions was lower than the current advisory level (i.e. 9 ng l^−1^ in Ontario, 10 ng l^−1^ in California), but the NDMA formation of water sample 16 was 9.8 ng l^−1^. This indicated that polyDADMAC should be replaced by other polymers when the bromide ion concentration is high in a water body.

**Table 3.** NDMA measurements for chloramination of coagulated water sample.

sample ID	NOM (mg l^−1^)	Br^−^ (μg l^−1^)	polyDADMAC (mg l^−1^)	Al_2_(SO_4_)_3_ (mg l^−1^)	NDMA (ng l^−1^)
1	0	0	0	0	<3
2	0	0	1	0	<3
3	0	0	1	20	<3
4	0	0	10	0	4.10 ± 1.11
5	0	0	10	200	4.32 ± 0.23
6	0	50	1	0	<3
7	0	500	10	0	7.83 ± 0.34
8	0	500	1	0	<3
9	1	0	1	0	<3
10	1	0	1	20	<3
11	10	0	10	0	4.67 ± 0.59
12	10	0	10	200	4.78 ± 1.03
13	10	50	10	200	5.31 ± 0.60
14	10	500	10	200	8.40 ± 1.28
15	10	50	10	0	6.78 ± 0.26
16	10	500	10	0	9.80 ± 0.10

## 4. Conclusion

According to the results of orthogonal experiment design, pH was the most important factor of NDMA formation from polyDADMAC, while NOM was the only factor that was not significant for residual DMA concentration. The highest NDMA formation of polyDADMAC occurred at pH 7, while the maximum residual DMA was at pH 6. The NDMA formation of polyDADMAC increased with bromide ion concentration, and the presence of bromide enhanced NDMA formation at all pH values due to the formation of a more reactive component, NHBrCl. The presence of NOM could reduce NDMA formation from polyDADMAC but the model experiments of coagulation through jar test showed the polyDADMAC still posed a serious threat for NDMA formation after coagulation. In simulated conditions in practice, overwhelming majority of NDMA formation from polyDADMAC was lower than the current advisory level, but polyDADMAC should be replaced by other polymers when the bromide ion concentration of water is high. The effects of bromide and NOM in NDMA formation from polyDADMAC degradation were first investigated in our experiment; further experiments on the effect of different monochloramine-to-bromide ratio and the effect of NOM in real water samples could be conducted.

## Supplementary Material

ShaojieJiang_tables_figures_texts_ESM.docx

## Data accessibility

Our data are available in Dryad: http://dx.doi.org/10.5061/dryad.878q6 [40].
