# Supplementary material for: Factors affecting N-nitrosodimethylamine formation from poly(diallyldimethyl-ammonium chloride) degradation during chloramination
Source: R Soc Open Sci. 2018 Aug 8;5(8):180025. doi: 10.1098/rsos.180025 (PMC6124131; doi:10.1098/rsos.180025)
Supplement: ShaojieJiang_tables_figures_texts_ESM.docx [file rsos180025supp1.docx]

Cover Page for Supporting Information:

Factors Affecting N-nitrosodimethylamine (NDMA) Formation From polyDADMAC Degradation During Chloramination

Siying Tan^a^; Shaojie Jiang^a,*^; Xiaoyu Li^a^; Qiuhong Yuan^b^

a School of Urban Construction and Environmental Engineering; Chongqing University, Chongqing, 400044, China

b Chongqing Qingze Water Quality Analysis Co., Ltd.; Chongqing, 401331, China

* Corresponding author: Tel.: +8615683628563; fax: +8615683628563; E-mail address: 20151701006@cqu.edu.cn (Shaojie Jiang).

**Total number of pages (including the cover page): 5 pages**

**This file contains 2 Tables, 2 Texts and 2 Figures.**

### Contents:

Text S1. Materials

Text S2. The process of jar test

Table S1. The factors and levels of orthogonal experimental design

Table S2. The analysis of variance in orthogonal experiment

Fig. S1. **Chemical Structure of polyDADMAC**

Fig. S2. Chlorine residuals as functions of pH after 24 h of chloramination

# Text S1. Materials

DMA hydrochloride (99%), Ammonium Chloride (99.5%), N, N-Diethyl-P-Phenylenediamine Sulfate (AR), potassium iodate (99.8%), potassium phosphate monobasic (99.5%), ethylenediamine tetraacetic acid disodium salt (99%), sodium acetate trihydrate (99%), sodium bicarbonate (99.5%), disodium hydrogenorthophosphate (99%), acetone (99.9%), benzenesulfonyl chloride (99%), potassium iodide (99.8%) were obtained from Kelong Chemical Industry. Sodium thiosulfate (99%), sodium hydroxide (96%), sodium hypochlorite (8.0-12.0%) and sodium bromide (99%) were purchased from Chongqing Boyi Chemical Industry. Sulfuric acid (95-98%) was obtained from Chongqing Chuandong Chemical Industry. Humic Acid (>70%) was purchased from Shanghai Yuanye Biological. Dichloromethane (99.9%) was obtained from TEDIA. Methanol (99.9%) was purchased from Fisher Scientific.

# Text 2. The process of jar test

Humic acid solution was prepared through following way: 1.0g humic acid was weighed and dissolved in 1000 ml 0.01 M NaOH solution for 24 h. The steps of jar test were: (1) polyDADMAC solution was added to the solution contained different concentration of humic acid or bromide in 1000 mL beakers; (2) stirred 2 min at 300 r/min (3) stirred 10 min at 70 r/min (4) settled for 30 min and supernatant was used to added performed monochloramine, buffered (10 mM phosphate buffer) at pH 7, and incubated in the dark at 25 °C for 24 h.

# Table S1 The factors and levels of orthogonal experimental design

| Factors | pH | Ammonia  (mg/L) | Bromide  (mM) | NOM (mg/L) | Monochloramine  (mg/L as Cl_2_/L) |
| --- | --- | --- | --- | --- | --- |
| Factor number | A | B | C | D | E |
| Level 1 | 5 | 0 | 0 | 0 | 2 |
| Level 2 | 6 | 0.1 | 0.05 | 0.25 | 4 |
| Level 3 | 7 | 0.4 | 0.1 | 0.5 | 6 |
| Level 4 | 8 | 1 | 0.2 | 1 | 8 |
| Level 5 | 9 | 1.5 | 0.3 | 2.5 | 10 |

# Table S2 The analysis of variance in orthogonal experiment

| Factor | Degree of Freedom | Critical Value of F-test | NDMA | | | DMA | |
| --- | --- | --- | --- | --- | --- | --- | --- |
|  |  |  | Sum of Deviation Squares | F Value | Sum of Deviation Squares | | F Value |
| pH | 4 | 6.39 | 1012069.50 | 7.92* | 57.64 | | 7.92* |
| Ammonia | 4 | 6.39 | 156517.27 | 1.23 | 70.68 | | 9.72* |
| Bromide | 4 | 6.39 | 727761.02 | 5.70 | 66.95 | | 9.20* |
| NOM | 4 | 6.39 | 336635.96 | 2.64 | 39.10 | | 5.37 |
| Monochloramine | 4 | 6.39 | 157204.96 | 1.23 | 46.79 | | 6.43* |
| Error |  |  | 127714.14 |  | 7.28 | |  |

* means significant factors

# Figure S1 **Chemical Structure of polyDADMAC**

# Figure S2 Chlorine residuals as functions of pH after 24 h of chloramination.


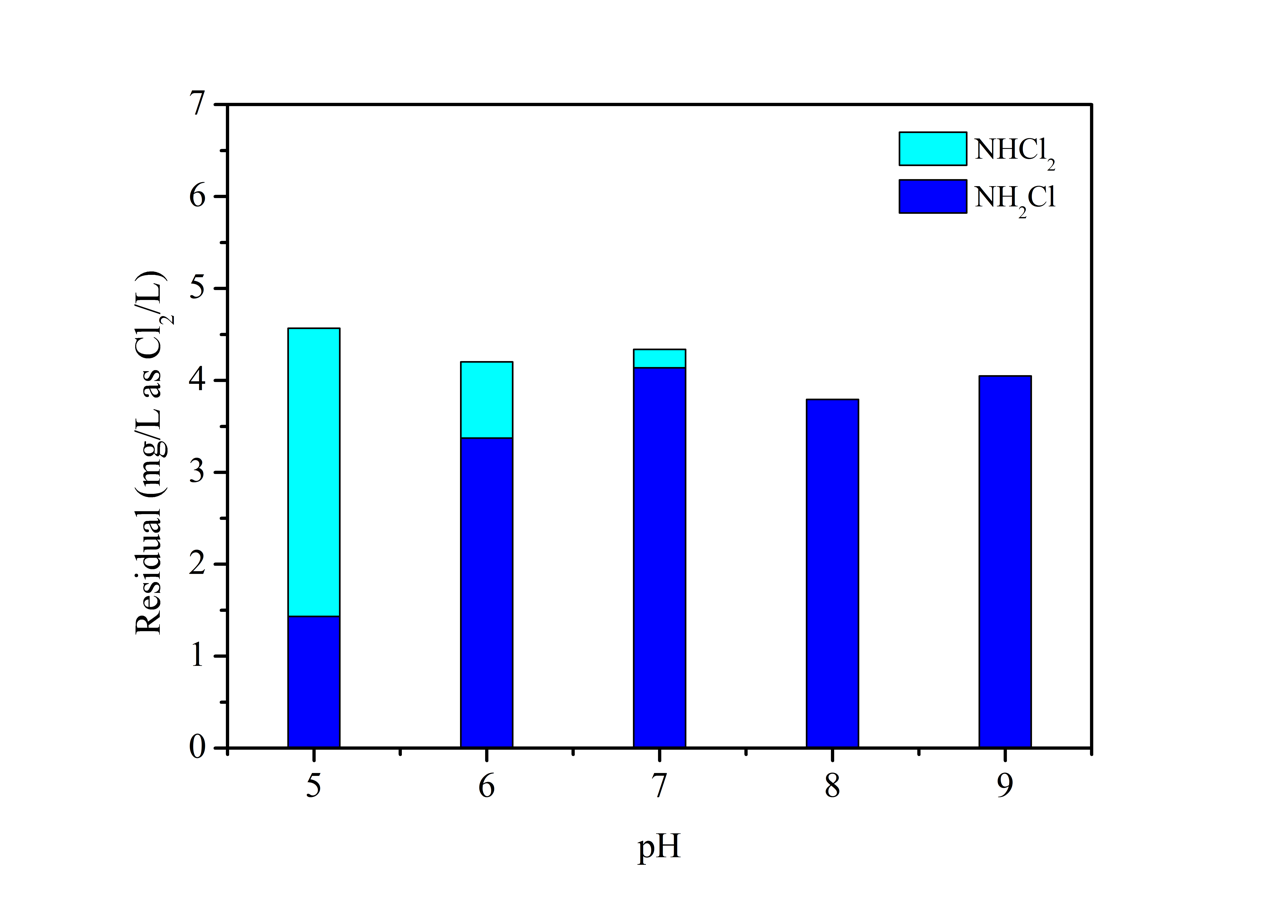


Chlorine residuals as functions of pH after 24 h of chloramination. Ten mg/L as active ingredient of polyDADMAC was reacted with 10 mg/L of Cl_2_/L of preformed monochloramine for 24h at 25 ℃ at pH 7 (10 mM phosphate buffer). Error bars represent one standard deviation of the measurement derived from the standard curve.
